# Validating the WHO maternal near miss tool: comparing high- and low-resource settings

**DOI:** 10.1186/s12884-017-1370-0

**Published:** 2017-06-19

**Authors:** Tom Witteveen, Hans Bezstarosti, Ilona de Koning, Ellen Nelissen, Kitty W. Bloemenkamp, Jos van Roosmalen, Thomas van den Akker

**Affiliations:** 1 Department of Obstetrics, Leiden University Medical Center, Albinusdreef 2, 2300 RC Leiden, the Netherlands; 2 Department of Obstetrics and Gynaecology, North Bristol NHS Trust, Southmead Hospital, Westbury-on-Trym, Bristol, BS10 5NB UK; 3 Wilhelmina Children’s Hospital Birth Center, University Medical Center Utrecht, Lundlaan 6, 3584 EA Utrecht, the Netherlands; 4 Athena Institute, VU University Amsterdam, De Boelelaan 1085, 1081 HV Amsterdam, the Netherlands

**Keywords:** Severe acute maternal morbidity, Maternal health, Maternal near miss, Maternal near miss-tool, World health organization, Delivery, Resource setting comparison, Organ dysfunction

## Abstract

**Background:**

WHO proposed the WHO Maternal Near Miss (MNM) tool, classifying women according to several (potentially) life-threatening conditions, to monitor and improve quality of obstetric care. The objective of this study is to analyse merged data of one high- and two low-resource settings where this tool was applied and test whether the tool may be suitable for comparing severe maternal outcome (SMO) between these settings.

**Methods:**

Using three cohort studies that included SMO cases, during two-year time frames in the Netherlands, Tanzania and Malawi we reassessed all SMO cases (as defined by the original studies) with the WHO MNM tool (five disease-, four intervention- and seven organ dysfunction-based criteria). Main outcome measures were prevalence of MNM criteria and case fatality rates (CFR).

**Results:**

A total of 3172 women were studied; 2538 (80.0%) from the Netherlands, 248 (7.8%) from Tanzania and 386 (12.2%) from Malawi. Total SMO detection was 2767 (87.2%) for disease-based criteria, 2504 (78.9%) for intervention-based criteria and 1211 (38.2%) for organ dysfunction-based criteria. Including every woman who received ≥1 unit of blood in low-resource settings as life-threatening, as defined by organ dysfunction criteria, led to more equally distributed populations. In one third of all Dutch and Malawian maternal death cases, organ dysfunction criteria could not be identified from medical records.

**Conclusions:**

Applying solely organ dysfunction-based criteria may lead to underreporting of SMO. Therefore, a tool based on defining MNM only upon establishing organ failure is of limited use for comparing settings with varying resources. In low-resource settings, lowering the threshold of transfused units of blood leads to a higher detection rate of MNM. We recommend refined disease-based criteria, accompanied by a limited set of intervention- and organ dysfunction-based criteria to set a measure of severity.

**Electronic supplementary material:**

The online version of this article (doi:10.1186/s12884-017-1370-0) contains supplementary material, which is available to authorized users.

## Background

One of the Millennium Development Goals was to reduce global maternal mortality in 2015 by three quarters as compared to the level of 1990 [1]. In the summer of 2015, the United Nations reported an estimated 45% decline (using data up to 2013), indicating that this target would not be fully met. In the meantime, new Sustainable Development Goals have been set, including the reduction of the maternal mortality ratio below 70 per 100.000 live births by 2030 [2]. Assessment of pregnant women with severe maternal outcome (SMO), comprised of maternal near miss (MNM) and maternal death (MD), may contribute to accelerating this morbidity and mortality reduction [3].

The World Health Organisation (WHO) has defined a MNM as a ‘woman who nearly died but survived a complication that occurred during pregnancy, childbirth or within 42 days of termination of pregnancy’ [3, 4]. WHO proposes a ‘MNM approach’ to monitor and improve quality of obstetric care using a tool that classifies women according to several (potentially) life-threatening conditions (Table 1) [4]. The classification is based on three different types of criteria: disease-, intervention- and organ dysfunction-based. If any of the organ dysfunction-based criteria are met, the MNM approach defines that case as ‘life-threatening’, and therefore MNM [5].

According to WHO, uniformity of this MNM classification should make it possible to compare the quality of obstetric care between different settings in different countries, which would be useful in improving health care delivery. However, in some low-resource settings application of the WHO MNM tool showed underreporting of life-threatening maternal morbidity. This may be due to lack of blood for transfusion, absence of laboratory diagnostics and poor clinical monitoring, which are all needed to identify MNM [6–8].

In a nationwide cohort, we previously found that also in the Netherlands, a high-resource setting, organ dysfunction-based criteria failed to identify almost 60% of women with severe acute maternal morbidities as MNM [9]. If these women, who were not detected as having had ‘life-threatening’ conditions, had attended obstetric care in low-resource settings the majority would likely have died.

Our previous studies have highlighted difficulties in finding universal criteria to identify MNM and raise questions about the applicability of the MNM tool in general, and its focus on organ dysfunction-based criteria in particular [6–9]. The objective of this study is to analyse merged data of one high- and two low-resource settings where this tool was applied and test whether the tool may be suitable for comparing SMO between these settings.

## Methods

In this current study, we used merged data available from SMO databases collected in the Netherlands, Tanzania and Malawi. Data for the Netherlands were extracted from a two-year nationwide cohort study (the LEMMoN-study), for Tanzania from a two-year cross-sectional study at Haydom Lutheran Hospital and for Malawi from a two-year study of maternal morbidity and mortality at Thyolo District Hospital (the ‘4 M-study’). A general description of the three study populations can be found in Table 1. Details and outcomes for these three cohorts have been published previously [7–10].

**Table 1:** WHO MNM tool groups and subcategories [4]

Group A	Severe complications/potentially life threatening conditions
A0	Severe postpartum hemorrhage
A1	Severe pre-eclampsia
A2	Eclampsia
A3	Sepsis or severe systemic infection
A4	Ruptured uterus
Group B	Critical interventions or intensive care unit admission
B0	Use of blood products (includes any blood transfusion)
B1	Interventional radiology (uterine artery embolization)
B2	Laparotomy (other than caesarean section)
B3	Admission to Intensive Care Unit
Group C	Organ dysfunction/life-threatening conditions
C0	Cardiovascular dysfunction: Shock, cardiac arrest (absence of pulse/ heart beat and loss of consciousness), use of continuous vasoactive drugs, cardiopulmonary resuscitation, severe hypoperfusion (lactate >5 mmol/l or >45 mg/dl), severe acidosis (pH <7.1)
C1	Respiratory dysfunction: Acute cyanosis, gasping, severe tachypnea (respiratory rate > 40 breaths per minute), severe bradypnea (respiratory rate < 6 breaths per minute), intubation and ventilation not related to anesthesia, severe hypoxemia (O2 saturation < 90% for ≥60 min or PAO2/FiO2 < 200)
C2	Renal dysfunction: Oliguria non-responsive to fluids or diuretics, dialysis for acute renal failure, severe acute azotemia (creatinine ≥300 μmol/ml or ≥3.5 mg/dl)
C3	Coagulation/ hematologic dysfunction: Failure to form clots, massive transfusion of blood or red cells (≥5 units), severe acute thrombocytopenia (<50,000 platelets/ml)
C4	Hepatic dysfunction: Jaundice in the presence of pre-eclampsia, severe acute hyperbilirubinemia (bilirubin >100 μmol/l or >6.0 mg/dl)
C5	Neurologic dysfunction: Prolonged unconsciousness (lasting ≥12 h)/coma (including metabolic coma), stroke, uncontrollable fits/status epilepticus, total paralysis
C6	Uterine dysfunction/ hysterectomy: Uterine hemorrhage or infection leading to hysterectomy

Women with SMO were included according to definitions established by the original studies (Table 2). We reassessed all cases in these three cohorts using the WHO MNM tool which defines MNM based on three different types of criteria: disease-, intervention- and organ dysfunction-based. Fourteen cases (0.4%) of the Dutch cohort were excluded due to insufficient data for application. All other 2538 SMO patients were assessed without the need for supplementation of any marker [9]. For the low-resource settings, identification of SMO did not only depend on relatively advanced laboratory tests, but could also happen on the basis of supplemented clinical markers as recommended by WHO [5].

**Table 2:** Demographics of the three study populations

	The Netherlands	Tanzania	Malawi
Study type	Prospective cohort	Prospective cohort	Prospective cohort
Period	2004-2006	2009-2011	2007-2009
Population	Nationwide	Haydom Lutheran Hospital	Thyolo District
Maternity units	98	1	29^b^
Reference area (km2)	41,526	51,000	1715
Live births^a^	375,657	9136	31,838
Deliveries^a^	371,021	9471	33,254

Data is shown in numbers

^a^During study period

^b^Including Thyolo District Hospital and 28 smaller, government, mission and private facilities

Data from the three studies were collected into a single database containing the following variables: age (<20, 20-35 and >35 years), parity (0, 1 and ≥2), units of blood given (0, 1, 2, 3, 4 and ≥5), duration of hospital stay, maternal mortality, and classification according to the three WHO MNM tool criteria groups (disease-, intervention- and organ dysfunction-based). If women had multiple conditions or interventions they were included into more than one criteria group, with each included criterion titled a separate ‘event’. Case fatality rates (CFR) were calculated for the corresponding populations.

All parameters were compared between each country’s population and those women who sustained life-threatening conditions as per WHO definition. Outcomes for the three countries were analysed individually and compared for differences. Finally, the life-threatening group was corrected by including every Tanzanian and Malawian woman (where giving five or more units is an exception even in life-threatening haemorrhage [11]) who received one unit or more of blood for transfusion. Maintaining five units of blood as an organ dysfunction criterion would imply that in settings where the availability of blood products is severely limited, fewer MNM cases are included.

Data were analysed using chi-square tests for categorical data and independent sample t-tests for numerical data. Statistical analysis was performed using SPSS statistics, version 20.0 (SPSS, Chicago, IL). All three initial studies had ethical approval and for present study anonymous data were used.

## Results

A total of 3172 women were analysed: 2538 (80.0%) from the Netherlands, 248 (7.8%) from Tanzania, and 386 (12.2%) from Malawi. General characteristics of all three populations are shown in Table 3. All parameters significantly differed between the three countries.

**Table 3:** Inclusion criteria of SMO used in the three study populations

The Netherlands	Tanzania	Malawi
ICU admissionAdmission to an ICU or coronary care unit, other than postoperative recovery	Clinical criteriaAcute cyanosis, gasping, respiratory rate > 40 or <6/min, shock, oliguria non responsive to fluids or diuretics, failure to form clots, loss of consciousness lasting >12H, cardiac arrest, stroke, uncontrollable fit/total paralysis, jaundice in the presence of pre-eclampsia	Uterine ruptureClinical symptoms or intrauterine foetal death that led to laparotomy, at which diagnosis was confirmed, laparotomy for uterine rupture after vaginal birth, rupture confirmed by autopsy or clinical symptoms with high suspicion of rupture after death
Uterine rupture Clinical symptoms that led to an emergency caesarean section, where uterine rupture was confirmed Peripartum hysterectomy or laparotomy for uterine rupture	Laboratory-based criteriaOxygen saturation < 90% for ≥60 minAcute thrombocytopenia (< 50,000 platelets/ml)	Eclampsia or severe pre-eclampsia with a maternal indication for termination of pregnancy
Eclampsia/HELLPHELLP syndrome only when accompanied by liver haematoma or rupture	Management-based criteria Admission to an ICU, hysterectomy following infection or haemorrhage, transfusion of ≥1 unit of blood, intubation and ventilation ≥60 min not related to anaesthesia, cardio-pulmonary resuscitation	Major obstetric haemorrhage(including from complicated abortions and ectopic pregnancies)Transfusion of units of ≥450 ml of blood or a haemoglobin level < 6 g/dl measured after vaginal bleeding or estimated blood loss of >1 l
Major obstetric haemorrhage (MOH)Transfusion of ≥4 units of packed cellsEmbolization or hysterectomy for MOH	Severe maternal complicationsEclampsia, sepsis or severe systemic infection, uterine rupture	Severe obstetric and non-obstetric peripartum infectionsAll infections for which iv antibiotics or iv anti-malarials were prescribed or surgical treatment was performed. Neoplasms resulting primarily from HIV-infections
MiscellaneousSMO cases to the opinion of the treating obstetrician, not to be included in group 1-4		Other complication ≥2 senior clinicians considered the condition as severe

*ICU* intensive care unit, *HELLP* haemolysis elevated liver enzymes and low platelets, *SMO* severe maternal outcome

After assessment with the WHO MNM tool, out of the 2538 Dutch women, 2308 (90.9%) fulfilled one or more disease-based criteria, 2116 (83.4%) any intervention-based criterion and 1024 (40.3%) any organ dysfunction-based criterion. In Tanzania there were 123 (49.6%) women fulfilling disease-based, 231 (85.9%) intervention-based, and 103 (41.5%) organ dysfunction-based criteria. For Malawi these numbers were 336 (87.0%), 175 (45.3%), and 84 (21.8%), respectively. The detection in the combined study population of 3172 women was 2767 (87.2%) women for disease-based, 2504 (78.9%) for intervention-based, and 1211 (38.2%) for organ dysfunction-based criteria. Only this final group sustained ‘life-threatening conditions’ according to WHO methodology. The CFRs were 48/2538 (1.9%) for the Netherlands, 32/248 (12.9%) for Tanzania and 46/386 (11.9%) for Malawi. Of these maternal deaths, 17 (35%) women in the Netherlands and 15 (33%) women in Malawi could not be identified as having had a ‘life-threatening’ condition. In Tanzania, all maternal deaths could be defined.

For the total population, analysis of the events detected by the WHO MNM tool subcategories is shown in Table 4. Postpartum haemorrhage (PPH) is the most commonly detected event among the disease-based criteria. Pre-eclampsia follows as an important second in the Netherlands, whereas in Tanzania and Malawi sepsis is more prominent. Giving blood products is the most frequent intervention and laparotomies (other than caesarean section) are more frequently performed in Malawi and Tanzania compared to the Netherlands. For the organ dysfunction-based criteria, coagulation or haematological dysfunction is the major reason for inclusion in the Netherlands, whereas in the low-resource settings this is cardiovascular dysfunction. Between countries all subcategories differed significantly except for the numbers of ruptured uterus (disease-based), admissions to ICU (intervention-based), and women who presented with renal dysfunction or ended up having hysterectomy (organ dysfunction-based).

**Table 4:** Basic characteristics of total study population

	Netherlands (*N* = 2538)	Tanzania (*N* = 248)	Malawi (*N* = 386)	*P*-value
Age (y)				
Data available	2512	248	384	
< 20	31 (1.2)	23 (9.3)	83 (21.6)	^b^
20-35	1945 (77.4)	187 (75.4)	267 (69.5)	^a^
> 35	536 (21.3)	38 (15.3)	34 (8.9)	^b^
Parity				
Data available	2388	227	377	
0	1258 (52.7)	52 (22.9)	83 (22.0)	^b^
1	867 (36.3)	30 (13.2)	56 (14.9)	^b^
≥ 2	263 (9.9)	145 (63.9)	238 (63.1)	^b^
Units of blood				
Data available	2461	248	371	
0	734 (29.8)	64 (25.8)	201 (54.2)	^b^
1	6 (0.2)	108 (43.5)	77 (20.8)	^b^
2	88 (3.6)	54 (21.8)	65 (17.5)	^b^
3	50 (2.0)	12 (4.8)	19 (5.1)	^b^
4	802 (32.6)	8 (3.2)	5 (1.3)	^b^
≥ 5	781 (31.7)	2 (0.8)	4 (1.0)	^b^
Mortality				
Data available	2538	248	386	
CFR	48 (1.9)	32 (12.9)	46 (11.9)	

Data is shown in numbers (percentage)

^a^= <0.05, ^b^ = <0.0001. CFR = case fatality rate

Among women with life-threatening conditions (as defined by the organ dysfunction-based criteria, Table 5), PPH is the most common event for inclusion in the Netherlands and Tanzania. In Malawi PPH, eclampsia, infection, and uterine rupture are almost equally represented. Eclampsia is significantly more common in both low-resource settings. Giving blood products is the commonest intervention-based criterion in the Netherlands and Malawi. In Tanzania this is ICU admission.

**Table 5:** WHO MNM tool inclusions of the total study population

Category	Subcategory	Events			
A: Disease		Netherlands (*N* = 2638)	Tanzania (*N* = 139)	Malawi (*N* = 394)	*P*-value
	0: PPH	1635 (62.0)	66 (47.5)	110 (27.9)	^b^
	1: Pre-eclampsia	414 (15.7)	8 (5.8)	20 (5.1)	^b^
	2: Eclampsia	242 (9.2)	15 (10.8)	69 (17.5)	^b^
	3: Sepsis	118 (4.5)	30 (21.6)	148 (37.6)	^b^
	4: Ruptured uterus	229 (8.7)	20 (14.4)	47 (11.9)	0.11
B: Intervention		Netherlands (*N* = 3030)	Tanzania (*N* = 334)	Malawi (*N* = 224)	
	0: Blood products	1743 (57.5)	184 (55.1)	165 (73.7)	^b^
	1: Int. radiology	111 (3.7)	N/A	N/A	
	2: Laparotomy	267 (8.8)	59 (17.7)	59 (26.3)	^b^
	3: Admission to ICU	909 (30.0)	91 (27.2)	N/A	0.78
C: Organ dysfunction		Netherlands (*N* = 1325)	Tanzania (*N* = 167)	Malawi (*N* = 96)	
	0: Cardiovascular	166 (12.5)	60 (35.9)	35 (36.5)	^b^
	1: Respiratory	115 (8.7)	35 (21.0)	13 (13.5)	^b^
	2: Renal	26 (2.0)	4 (2.4)	1 (1.0)	0.21
	3: C/H	845 (63.8)	16 (9.6)	4 (4.2)	^b^
	4: Hepatic	27 (2.0)	3 (1.8)	11 (11.5)	^a^
	5: Neurologic	33 (2.5)	33 (19.8)	11 (11.5)	^b^
	6: Hysterectomy	113 (8.5)	16 (9.6)	21 (21.9)	0.29

Data is shown in numbers (percentage)

*PPH* postpartum haemorrhage, *ICU* intensive care unit, *Int. radiology* interventional radiology, *C/H* coagulation/haematological, *N/A* not applicable

^a^= <0.05, ^b^ = <0.0001

After correction for any blood transfusion in the low-resource settings the life-threatening group changed (Table 5). First, the MNM tool now identified 1458 (46.0%) women with organ dysfunction, instead of 1205 (38.2%). In addition, blood transfusion became a more frequent inclusion criterion in the low-resource settings as compared to the Dutch setting, and ‘coagulation or hematologic dysfunction’ was now equally represented in each setting. When including any blood transfusion, the position of PPH as major contributor to severe acute maternal morbidity becomes more prominent in Tanzania and Malawi (36.4% and 24.4% raised to 53.2% and 42.6%).

The WHO MNM tool inclusions and general characteristics of women with life-threatening conditions (before and after correction for blood transfusion) can be seen in Tables 6 and 7. In comparison with the total study population (Table 3) higher CFRs are seen among women with life-threatening conditions, and among women in low-resource settings.

**Table 6:** WHO MNM tool inclusions of the (corrected) life-threatening population

Category	Subcategory	Events						
A: Disease		Netherlands (*N* = 1132)	Tanzania (*N* = 77)	Corrected (*N* = 124)	Malawi (*N* = 86)	Corrected (*N* = 216)	*P*-value	Corrected
	0: PPH	822 (72.6)	28 (36.4)	66 (53.2)	21 (24.4)	92 (42.6)	^a^	^a^
	1: Pre-eclampsia	160 (14.1)	3 (3.9)	5 (4.0)	5 (5.8)	7 (3.2)	^a^	^a^
	2: Eclampsia	52 (4.6)	15 (19.5)	15 (12.1)	21 (24.4)	25 (11.6)	^a^	^a^
	3: Sepsis	52 (4.6)	20 (26.0)	23 (18.5)	21 (24.4)	56 (25.9)	^a^	^a^
	4: Ruptured uterus	46 (4.1)	11 (14.3)	15 (12.1)	18 (20.9)	36 (16.7)	^a^	^a^
B: Intervention		Netherlands (*N* = 1725)	Tanzania (*N* = 153)	(*N* = 315)	Malawi (*N* = 66)	(*N* = 215)		
	0: Blood products	895 (51.9)	59 (38.6)	184 (58.4)	43 (65.2)	165 (76.7)	^a^	^a^
	1: Interv. radiology	96 (5.6)	N/A	N/A	N/A	N/A		
	2: Laparotomy	197 (11.4)	27 (17.6)	50 (15.9)	23 (34.8)	50 (23.3)	0.06	0.21
	3: Admission to ICU	537 (31.1)	67 (43.8)	81 (25.7)	N/A	N/A		
C: Organ dysfunction		Netherlands (*N* = 1325)	Tanzania (*N* = 167)	(*N* = 337)	Malawi (*N* = 96)	(*N* = 257)		
	0: Cardiovascular	166 (12.5)	60 (35.9)	60 (17.8)	35 (36.5)	35 (13.6)	^a^	^a^
	1: Respiratory	115 (8.7)	35 (21.0)	35 (10.4)	13 (13.5)	13 (5.1)	^a^	^b^
	2: Renal	26 (2.0)	4 (2.4)	4 (1.2)	1 (1.0)	1 (0.4)	0.51	0.16
	3: C/H	845 (63.8)	16 (9.6)	186 (55.2)	4 (4.2)	165 (64.2)	^a^	0.70
	4: Hepatic	27 (2.0)	3 (1.8)	3 (0.9)	11 (11.5)	11 (4.3)	^a^	^b^
	5: Neurologic	33 (2.5)	33 (19.8)	33 (9.8)	11 (11.5)	11 (4.3)	^a^	^a^
	6: Hysterectomy	113 (8.5)	16 (9.6)	16 (4.7)	21 (21.9)	21 (8.2)	^a^	0.20

Data is shown in numbers (percentage)

*PPH* postpartum haemorrhage, *ICU* intensive care unit, C/H = coagulation/haematological, *N/A* not applicable

^a^= <0.0001, ^b^ = <0.05

**Table 7:** Basic characteristics of the (corrected) life-threatening population

	Netherlands (*N* = 1024)	Tanzania (*N* = 103)	Corrected (*N* = 228)	Malawi (*N* = 84)	Corrected (*N* = 206)	*P*-value	Corrected
Age (y)							
Data available	1019	103	228	84	205		
< 20	11 (1.1)	15 (14.6)	22 (9.6)	16 (19.0)	29 (14.1)	^a^	0.15
20-35	760 (74.6)	75 (72.8)	170 (74.6)	54 (70.2)	157 (76.2)	0.71	0.69
> 35	248 (24.3)	13 (12.6)	36 (15.8)	9 (10.7)	19 (9.2)	^a^	^b^
Parity							
Data available	967	93	208	81	202		
0	514 (53.2)	28 (30.1)	47 (22.6)	19 (23.5)	32 (15.8)	^a^	0.08
1	333 (32.5)	10 (10.8)	27 (13.0)	9 (11.1)	28 (13.6)	^a^	0.79
≥ 2	120 (12.4)	55 (59.1)	134 (64.4)	53 (65.4)	142 (70.3)	^a^	0.21
Units of blood							
Data available	1000	103	228	82	202		
0	123 (12.3)	44 (42.7)	44 (19.3)	39 (47.6)	49 (24.3)	^a^	0.21
1	6 (0.6)	22 (21.4)	108 (47.4)	14 (17.1)	64 (31.7)	^a^	^c^
2	23 (2.3)	25 (24.3)	54 (23.7)	17 (22.1)	62 (30.7)	^a^	0.10
3	16 (1.6)	6 (5.8)	12 (5.3)	5 (6.1)	17 (8.4)	^a^	0.19
4	88 (8.8)	4 (3.9)	8 (3.5)	3 (3.7)	5 (2.5)	0.07	0.53
≥ 5	744 (74.4)	2 (1.9)	2 (0.9)	4 (4.9)	4 (2.0)	^a^	0.33
Mortality							
Data available	1024	103	228	84	206		
CFR	31 (3.0)	32 (31.1)	32 (14.0)	21 (25.0)	28 (13.6)		

Data is shown in numbers (percentage)

*CFR* case fatality rate

^a^= <0.0001, ^b^ = <0.05, ^c^ = <0.01

## Discussion

Our results indicate that the WHO MNM tool, in its current form, is not useful for comparison between different resource settings. Detection differs between high- and low-income countries and organ dysfunction-based criteria detect only 38.2% of all women with SMO as defined by the three cohort studies.

Moreover, in cases of maternal *mortality* and based on the specified criteria, organ dysfunction could not be identified from the medical records in 17 out of 48 cases (35%) in the Netherlands and 15 out of 46 cases (33%) in Malawi. We believe that a revision of the WHO MNM tool and specifically the organ dysfunction-based criteria is needed to enable meaningful comparison between different resource settings.

A recent study by Menezes et al. states that the WHO criteria perform well [12]. In this study, conducted in two Brazilian reference hospitals, 77 out of 1196 (6.4%) women were identified as having life-threatening conditions based on the WHO MNM tool, compared to 33.8% and 80.2% by using Waterstone’s or other literature-based criteria respectively. However, the authors do not clarify why the other 1119 (93.6%) women did not sustain MNM conditions or why these pregnant women did not ‘nearly die, but survived’ (according to WHO MNM definition). The reason for this omission appears that the current WHO criteria are mistakenly seen as the ‘gold standard’ for evaluation of severe maternal morbidity.

The underestimation of severe maternal outcome when applying the WHO MNM tool in its current form remains an important issue. Overall, disease-based criteria show the highest detection of SMO (87.2%) in each type of setting. An explanation for the low detection rate (49.6%) in the Tanzanian population could be the local SMO criteria used in that study. For example, this led to fewer women with PPH (according to the WHO MNM definition of blood loss above one liter) in this cohort, as PPH as such was no separate inclusion criterion in the Tanzanian cohort (in contrast with Malawi) and women were only included if they had received blood transfusion. The intervention-based criteria detected 78.9% of all SMO cases. An explanation for the low detection (45.3%) in the Malawian population is the absence of interventional radiology and an ICU. Both disease-based and intervention-based criteria show higher SMO detection in each setting compared to organ dysfunction-based criteria. The CFRs of the potentially life-threatening populations (fulfilling only disease-based criteria) in low-resource settings remain high (Tanzania 13/123, 10.6%; Malawi 35/336, 10.4% versus 23/2308, 1.0% in the Netherlands). This implies that there is hardly any ‘over-inclusion’ in such settings and that these women should be picked up as SMO in the ‘potentially life-threatening phase’ of their conditions.

The lack of laboratory and clinical diagnostics for detecting organ dysfunction explains underreporting in low-resource settings [6–9]. Similar detection rates for Tanzania and the Netherlands may seem contradictory because advanced technology in the highly resourced Dutch setting would be expected to lead to a higher detection of SMO. An explanation could be found in the supplemented clinical criteria (such as acute cyanosis, gasping, loss of consciousness etc.) as part of the local Tanzanian inclusion criteria (Table 1). These compensate the lack of extensive intensive care monitoring needed for detection by organ dysfunction-based criteria. This would also explain the low detection numbers in Malawi due to the mainly disease- and intervention-based local inclusion criteria.

Different criteria for SMO used in the three cohorts are the most important limitation of this study. SMO cases, as identified differently by local criteria, are being compared according to a single WHO MNM tool. The consequence may be an underestimation of SMO in low-resource settings as Tanzania and Malawi due to limited available diagnostics. However, this limitation also stresses the fact that application of the WHO MNM tool may differ in different contexts.

Another major issue is that, although WHO uses a threshold of five units, there is no consensus about the number of units of blood transfused, which identifies organ dysfunction [6–9]. After including every woman in a low-resource setting who received even one unit of blood, results show a more equally distributed ‘life-threatening group’ in all settings, emphasizing that the shortage of blood for transfusion remains a large problem in many low-resource settings [13]. Also, SMO detection rate increased from 38.2% to 46.0% of all SMO cases. This 7.8% increase consists of 228 Tanzanian women (91.9%) and 206 Malawian women (53.4%). This leads to a more realistic comparison between high- and low-resource settings, because PPH is an important cause of SMO and lack of blood compounds this problem [11, 14]. Unfortunately, this is also due to unwillingness and impossibility of relatives to donate, and inadequacy or lack of blood bank storage facilities and transport [6, 7, 11, 15].

Although it is clear that there is an urgent need for monitoring health care delivery in both high- and low-resource settings, it remains difficult to determine which set of criteria should be used. In our opinion, disease-based criteria remain important in all settings, since detection rate is high and does not depend on local protocols. In contrast, for the same reason, intervention-based criteria (such as ICU admission) are of limited use. To prevent ‘over-inclusion’ for disease-based criteria, especially in high-income countries, more strict operational definitions (such as the blood loss threshold defining ‘severe postpartum haemorrhage’) are needed. For low-resource settings, supplemented clinical markers such as gasping, oliguria or jaundice could be included. Also, the threshold of received units of blood should be lowered for organ dysfunction-based criteria [8].

## Conclusions

In conclusion, we have shown that applying solely organ dysfunction-based criteria may lead to underreporting of SMO. Therefore, a tool based on defining MNM only upon establishing organ failure is of limited use for comparing settings with varying resources. It is important to enact the discussion and eventually reach consensus for a tool that is usable in all resource settings and detects the highest percentage of the actual rate of SMO. We recommend refined disease-based criteria, accompanied by a limited set of (intervention- and organ dysfunction-based) criteria to set a measure of severity. We believe that with these adjustments, the MNM tool may be more valuable and could ultimately lead to more comparable assessments of the quality of obstetric health care across different settings.

## Additional file

Additional file 1: Details of local ethics committees. (DOCX 62 kb)

## Abbreviations

CFR: Case fatality rate
ICU: Intensive care unit
MD: Maternal death
MNM: Maternal near miss
PPH: Postpartum haemorrhage
SMO: Severe maternal outcome
WHO: World health organisation
