# Supplementary material for: Validating the WHO maternal near miss tool: comparing high- and low-resource settings
Source: BMC Pregnancy Childbirth. 2017 Jun 19;17:194. doi: 10.1186/s12884-017-1370-0 (PMC5477239; doi:10.1186/s12884-017-1370-0)
Supplement: Additional file 1: — Details of local ethics committees. (DOCX 62 kb) [file 12884_2017_1370_MOESM1_ESM.docx]

**Supplemental file: details of local ethics committees**

Netherlands

Medical ethics committee of the Leiden University Medical Centre, Leiden, the Netherlands.

Reference: P04-020

Tanzania

National Institute for Medical Research, Dar es Salaam, Tanzania.

Reference: NIMR/HQ/R.8a/Vol.IX/1247

Commission for Science and Technology, Dar es Salaam, Tanzania.

Reference: 2012-56-NA-2011-201

Medical ethics committee of the VU University Medical Centre, Amsterdam, the Netherlands.

Reference: 2011/389

Malawi

National Health Sciences Research Committee from the Ministry of Health, Lilongwe, Malawi.

The District Health Office of the Ministry of Health, Lilongwe, Malawi.
